# Prevalence of Complementary and Alternative Medicine Use in Fracture Patients: a Tertiary Trauma Center Observation

**DOI:** 10.1007/s44197-025-00496-6

**Published:** 2025-12-30

**Authors:** Abdulrahman Alaseem, Sarah Alflaij, Amjad Albaroudi, Sarah Alaidarous, Banan Alqady, Yazeed Alsanad, Nizar Algarni, Ibrahim Alshaygy, Waleed Albishi

**Affiliations:** 1 Department of Orthopedic Surgery, College of Medicine, King Saud University, Riyadh, Saudi Arabia; 2 College of Medicine, King Saud University, Riyadh, Saudi Arabia; 3 Third Health Cluster, Riyadh, Saudi Arabia; 4 Department of Emergency Medicine, King Saud University, College of Medicine, Riyadh, Saudi Arabia; 5 Department of Orthopedic Surgery, Security Forces Hospital, Riyadh, Saudi Arabia

**Keywords:** Complementary and alternative medicine (CAM), Fracture patients, Fracture healing, Orthopedics, Saudi arabia

## Abstract

**Background:**

Complementary and alternative medicine (CAM) treatment practices are frequently utilized by patients alongside conventional modern medicine. CAM options are commonly practiced by orthopedic trauma patients and are usually driven by cultural and sometimes religious beliefs. This study aims to evaluate the prevalence, practices and influencing factors of CAM use among orthopedic patients.

**Methodology:**

An observational cross-sectional survey study was carried out at the Orthopedic Department fracture clinics in a tertiary trauma center in Riyadh, Saudi Arabia. Participants were recruited from orthopedic fracture clinics and requested to complete a questionnaire survey.

**Results:**

Among the 356 surveyed fracture patients, 49.4% reported using CAM. Of these CAM users, 20.5% patients used CAM for the first time after being diagnosed with fracture, while 79.5% of patients had previously utilized CAM and increased their usage after fracture diagnosis. CAM practices were significantly associated with older age, joint reconstructive fracture treatment, and reported disability/dependence. Fenugreek was the most commonly consumed type by 81% of CAM users. The key motivations for CAM use were the perception of enhancing the fracture healing process and maintaining good health. Notably, 61.5% of CAM users reported experiencing improvements in their physical health, while 9.0% of the users experienced adverse effects such as weight gain and joint pain.

**Conclusion:**

Our study shed a light on the high prevalence of CAM usage among fracture patients, with Fenugreek being the most popular. Potential influencing factors for CAM practices were investigated including demographic, socioeconomic as well as fracture-related factors. Despite its high prevalence, CAM use is often not discussed with the treating physicians, therefore, increased physician awareness and enhanced patient communication about CAM practices are essential. Further investigations are required to explore CAM adverse effects as well as impact on fracture healing and general health.

## Introduction

 Complementary and alternative medicine (CAM) refers to practices that differ from conventional modern medicine, including the use of herbs, dietary supplements, mind-body techniques, and traditional healing methods such as spiritual healing and traditional Chinese medicine [1, 2]. Worldwide, CAM therapies are gaining popularity as complementary approaches for managing chronic illnesses, enhancing well-being, and improving quality of life. Despite their increasing prevalence, the efficacy and safety of many CAM practices remain underexplored, necessitating further investigation to better understand their potential advantages and risks [2, 3]. In Saudi Arabia, the widespread adoption of CAM is deeply rooted in cultural diversity and religious beliefs [4].

Among patients with musculoskeletal conditions, particularly fractures, CAM practices are often perceived as beneficial adjuncts to conventional treatments. Previous studies suggest that certain CAM modalities and dietary supplements may promote bone healing and reduce inflammation, potentially accelerating recovery and minimizing the risk of recurrent fractures [5, 6]. Similarly, A recent study by Gunawan et al. (2024) highlighted that CAM can effectively reduce pain intensity in fracture patients, proposing their potential as adjuncts to conventional care [7]. A recent systematic review also addressed the wide variety of complementary and alternative medicine techniques used to promote bone fracture healing [8]. However, integrating CAM with conventional therapies requires cautious consideration, as limited evidence supports the clinical efficacy and safety of these practices in the context of orthopedic injuries [2, 6]. Fenugreek (Trigonella foenum-graecum L.) is a traditionally recognized remedy deeply rooted in the healing tradition of the Gulf and Saudi Arabia. Its callus-promoting effect was highlighted from a recent Saudi case report of a humeral shaft fracture in Saudi Arabia [9], indicating community reliance upon herbal modes of musculoskeletal treatment with very high use of herbal and complementary medicine by Saudi patients [10]Within this cultural context, fenugreek use in fracture patients both persists in traditional practice and represents a new field of scientific exploration.

The use of complementary and alternative medicine (CAM) can be influenced by various factors, including demographic characteristics and the specifics of a patient’s medical condition. For example, studies have shown that age and gender often play a role in whether individuals choose to use CAM [11]. Additionally, clinical factors such as the severity of symptoms or the type of treatment they receive can also impact the likelihood of CAM usage. These influences highlight the complex interplay between personal and medical factors in shaping CAM practices.

Despite the frequent use of CAM by patients with musculoskeletal conditions, there is a notable scarcity of literature investigating the prevalence and patterns of CAM usage specifically among fracture patients. This gap is particularly relevant in Saudi Arabia, where traditional practices like fenugreek consumption are commonly believed to support fracture healing. Moreover, the role of demographic, socioeconomic, and clinical factors in shaping CAM utilization in this population remains underexplored. This study aims to address this gap by evaluating the prevalence, patterns, and determinants of CAM usage among fracture patients at a tertiary trauma center in Riyadh, Saudi Arabia. Through a cross-sectional analysis, we seek to highlight the motivations for CAM practices, their perceived benefits, and the extent of patient disclosure to healthcare providers. By providing valuable insights into this prevalent yet understudied phenomenon, our study aims to inform clinical practitioners and foster improved physician-patient communication regarding CAM usage.

## Materials and Methods

An observational cross-sectional study was carried out in January and February 2024 at the Orthopedic Department fracture clinics in a tertiary trauma university hospital in Riyadh, Saudi Arabia, which has a high volume of musculoskeletal trauma cases. Following obtaining the institutional review board (IRB) approval, a convenience sampling was used, where all patients in the waiting room of fracture clinics were interviewed physically, and patients scheduled in the virtual clinics’ appointment list were interviewed via telephone. As convenience sampling was adopted, the possibility of selection bias must be acknowledged, and the generalizability of findings may be limited.

Participants included patients with various fracture anatomical locations, characteristics, timing, management, and prior hospitalization history. After being consented to participate voluntarily and ensuring their confidentiality, they were requested to answer a survey questionnaire focusing on the prevalence and patterns of CAM use in fracture patients. For participants interviewed via telephone, verbal informed consent was explicitly obtained prior to the interview, in line with the IRB protocol, and confidentiality was strictly maintained.

The required sample size was calculated using the formula n = (Z^2^⋅p⋅(1 − p))/E^2^, where Z = 1.96 (95% confidence interval), *p* = 0.5 (estimated proportion for maximum variability), E = 0.05 (margin of error). This resulted in a required sample size of 385 participants. To account for potential non-responses, we increased the target sample size to 470 participants. This ensured that, even with a response rate of 75.7%, the final sample size of 356 participants was sufficient for statistical analysis. A total of 470 participants were recruited and a structured questionnaire was distributed which involved three main parts: demographic data, fracture-related details, and practices and perceptions of CAM. In the first part, participants were asked about their age, gender, marital status, nationality, religion, education level and income. The second part involved inquiries relevant to the fracture characteristics such as location, duration, treatment received, pain score and other associated symptoms. The third part explored CAM usage, including types, sources, patterns of use, motivations, benefits, frequency, adverse effects, and the influencing factors for considering CAM utilization, as well as physician awareness and perceptions of their use. The questionnaire items were adapted from prior CAM literature and piloted for clarity among a small group of fracture patients; however, no formal psychometric validation was undertaken, which should be considered a methodological limitation.

Data were analyzed using SPSS 29.0.2.0 version (IBM Inc., Chicago, USA) statistical software. Descriptive statistics (frequencies, percentages, median, Interquartile range, mean and standard deviation) were used for categorical and quantitative variables. The Mann-Whitney Test was used to compare the median age between CAM users and non-users to evaluate significant differences, as the age data was continuous and not normally distributed. For categorical variables, Pearson’s Chi-Square test was used for nominal data, while the Linear-by-Linear Association test was applied to ordinal data. A p-value of ≤ 0.05 and 95% confidence intervals were used to report the statistical significance and precision of the result.

## Results

Out of 470 eligible patients, 54 refused to participate and 60 did not answer, yielding 356 participants included in the final study analysis with a response rate of 75.7%. The majority of patients were males 224 (62.9%), while the mean (± SD) age was 29.0 (± 21.0) years with an overall age range from 8 to 85 years. Regarding education level, most respondents either hold a bachelor’s degree (37.5%) or a high school certificate (37.2%). Additionally, only 178 (50%) participants disclosed their income, of which the majority 83 (46.6%) reported an income exceeding 8000 Saudi Riyals (SAR).

**Fig. 1:**
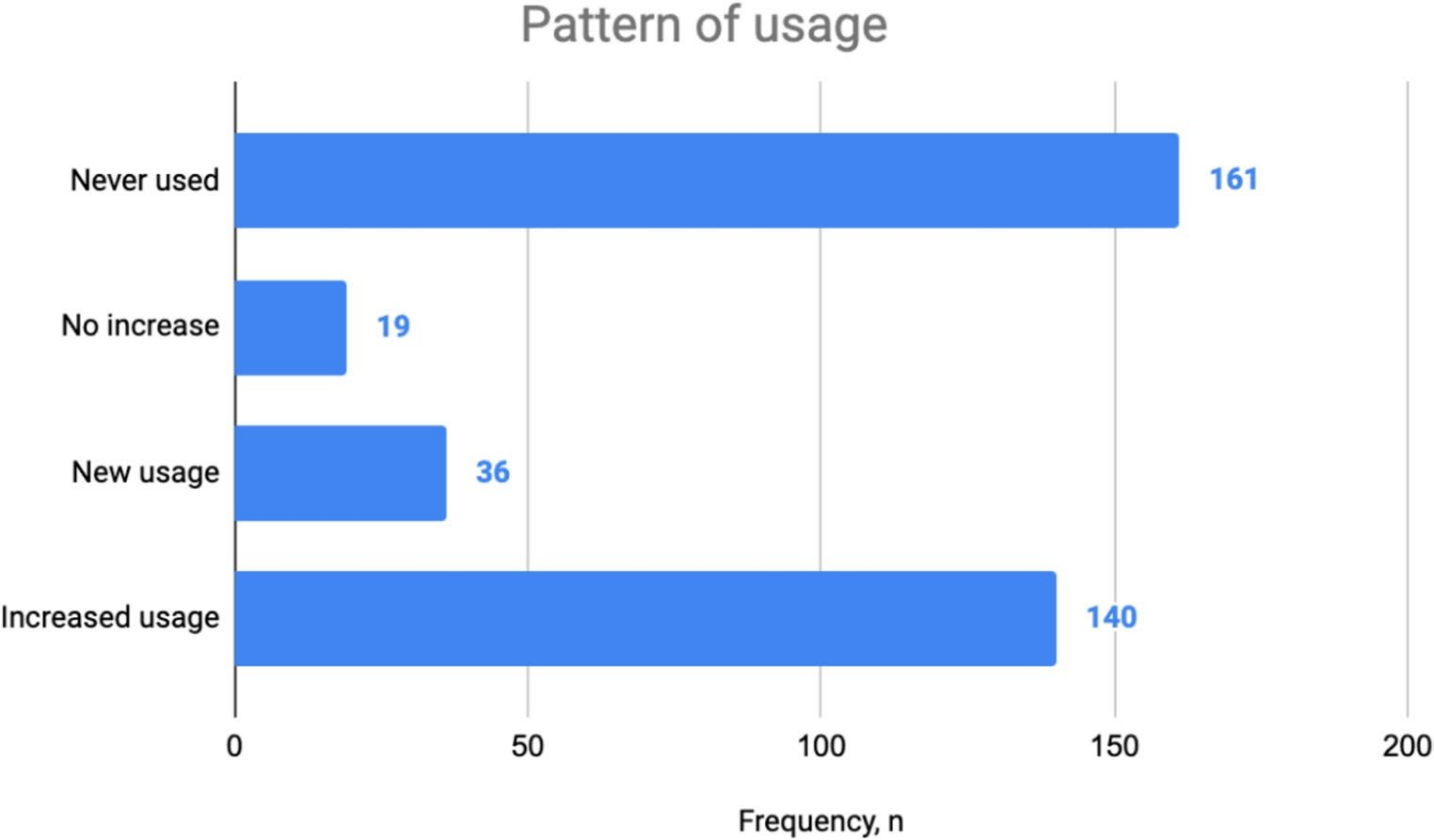
Pattern of usage of CAM among fracture patients, Riyadh, Saudi Arabia, 2024

An overall number of 176 (49.4%) surveyed fracture patients have utilized CAM practices, 36 (10.1%) of which started using CAM after sustaining a fracture, while 140 (39.3%) were previous CAM users and they increased usage pattern after being fractured (Figure. 1).

The demographic and socioeconomic characteristics for CAM users (*n* = 176) are shown in Table 1 with mean age for CAM users is 35.6 years in comparison to 30.3 years among non-users (*p* < 0.001). CAM usage was more common among female patients while males were less likely to utilize CAM relative to their male cohort although the difference was not statistically significant (*p* = 0.089). Non-Saudi patients seem to practice CAM less frequently (3 out of 12) when compared to Saudi nationals (173 out of 344) (*p* = 0.085). Neither the educational level nor the economic status exhibited statistically significant influence on CAM practices.

**Table 1:** Demographic and socioeconomic caharcteristics of CAM users

Demographic	CAM users (*N* = 176)	Non-users (*N* = 180)	Total (*N* = 356)	*P* value
Age^e^ (median ± IQR^d^)	33.5 ± 24.0	26.0 ± 17.0	29.0 ± 21.0	**< 0.001** ^**a**^
Gender				
Female	73 (41.5)	59 (32.8)	132 (37.1)	0.089 ^b^
Male	103 (58.5)	121 (67.2)	224 (62.9)	
Marital Status				
Married	71 (40.30)	58 (32.20)	129 (36.20)	0.110 ^b^
Single	88 (50.00)	112 (62.20)	200 (56.20)	
Divorced	11 (6.30)	7 (3.90)	18 (5.10)	
Widowed	6 (3.40)	3 (1.70)	9 (2.50)	
Nationality				
Non-Saudi	3 (1.70)	9 (5.00)	12 (3.40)	0.085 ^b^
Saudi	173 (98.30)	171 (95.00)	344 (96.60)	
Education				
Illiterate	7 (4.00)	4 (2.20)	11 (3.10)	0.734 ^c^
Primary school	6 (3.40)	5 (2.80)	11 (3.10)	
Elementary school	15 (8.50)	18 (10.10)	33 (9.30)	
High school	61 (34.70)	71 (39.70)	132 (37.20)	
Diploma	5 (2.80)	10 (5.60)	15 (4.20)	
Bachelor	73 (41.50)	60 (33.50)	133 (37.50)	
Postgraduate	9 (5.10)	11 (6.10)	20 (5.60)	
Income				
< 3000 SAR	12 (12.20)	17 (21.30)	29 (16.30)	0.642 ^c^
3000–8000 SAR	42 (42.90)	24 (30.00)	66 (37.10)	
> 8000 SAR	44 (44.90)	39 (48.80)	83 (46.60)	

a Mann-Whitney Test

b Pearson Chi-Square

c Linear-by-Linear Association

d Interquartile range

e A Kolmogorov-Smirnov Test showed a significant departure from normality, W(356) = 0.12, *p* < 0.001

Table 2 presents the fracture characteristics for CAM users and Non-users including fracture age, location, management and symptoms. Lower limb fractures were the most common location in both groups and the majority were managed nonoperatively with cast. CAM usage among fracture patients with disability/dependence (*p* = 0.026) as well as patients treated with joint reconstructive surgery (*p* = 0.017) or rest (*p* = 0.006) was statistically significant. Stratification by fracture site revealed that CAM utilization was more frequent among patients with lower limb fractures compared with those with upper limb injuries (57.9% vs. 36.8%, *p* < 0.05).

**Table 2:** Fracture characteristics, management, and symptoms

Demographic	CAM users (*N* = 176)	Non-users (*N* = 180)	Total (*N* = 356)	*P* value
Fracture Age				
< 1 month	16 (9.10)	15 (8.30)	31 (8.70)	0.580^c^
1–3 months	50 (28.40)	47 (26.10)	97 (27.20)	
> 3 months	110 (62.50)	118 (65.60)	228 (64.00)	
Fracture Location				
Lower Limb	110 (57.90)	114 (60.60)	224 (59.30)	0.871^b^
Upper Limb	70 (36.80)	64 (34.00)	134 (35.40)	0.412^b^
Spine	9 (4.70)	9 (4.80)	18 (4.80)	0.961^b^
Ribs	1 (0.50)	1 (0.50)	2 (0.50)	0.987^b^
Management				
Internal fixation	76 (36.00)	64 (29.50)	140 (32.70)	0.141^b^
External fixation	6 (2.80)	3 (1.40)	9 (2.10)	0.295^b^
Joint reconstruction	10 (4.70)	2 (0.90)	12 (2.80)	**0.017**^b^
Cast	87 (41.20)	93 (42.90)	180 (42.10)	0.673^b^
Immobilization	20 (9.50)	26 (12.00)	46 (10.70)	0.386^b^
Rest	12 (5.70)	29 (13.40)	41 (9.60)	**0.006**^b^
Symptoms				
Pain	171 (41.10)	179 (45.90)	350 (43.40)	0.094^b^
Stiffness	77 (18.50)	71 (18.20)	148 (18.40)	0.410^b^
Weakness	84 (20.20)	75 (19.20)	159 (19.70)	0.250^b^
Disability/dependence	84 (20.20)	65 (16.70)	149 (18.50)	**0.026**^b^
Pain Scale out 10	7.7 ± 2.8	7.9 ± 2.4	7.8 ± 2.6	0.367^c^

a Mann-Whitney Test

b Pearson Chi-Square

c Linear-by-Linear Association

**Fig. 2:**
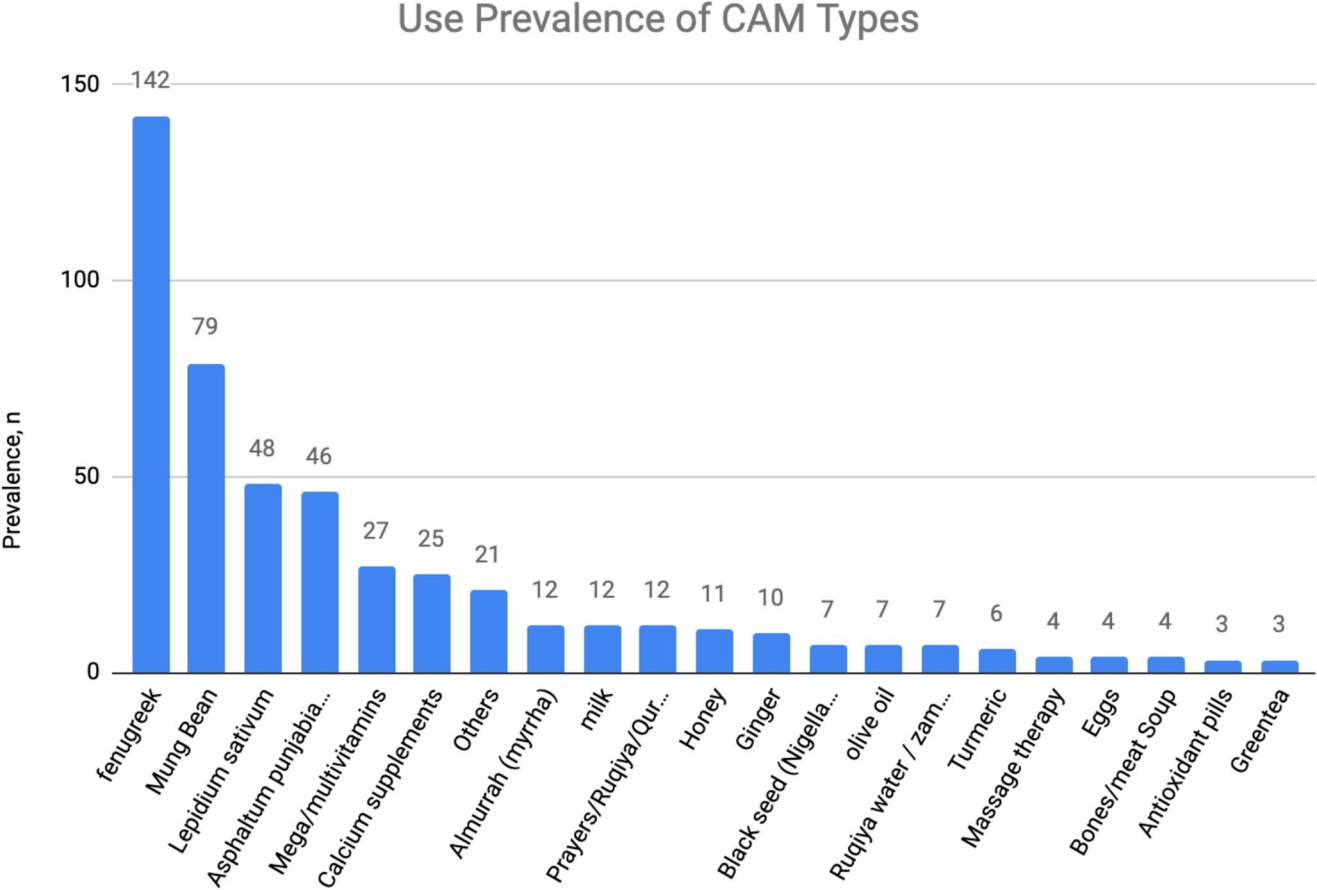
Prevalence of different types of CAM utilized by fracture patients

**Fig. 3:**
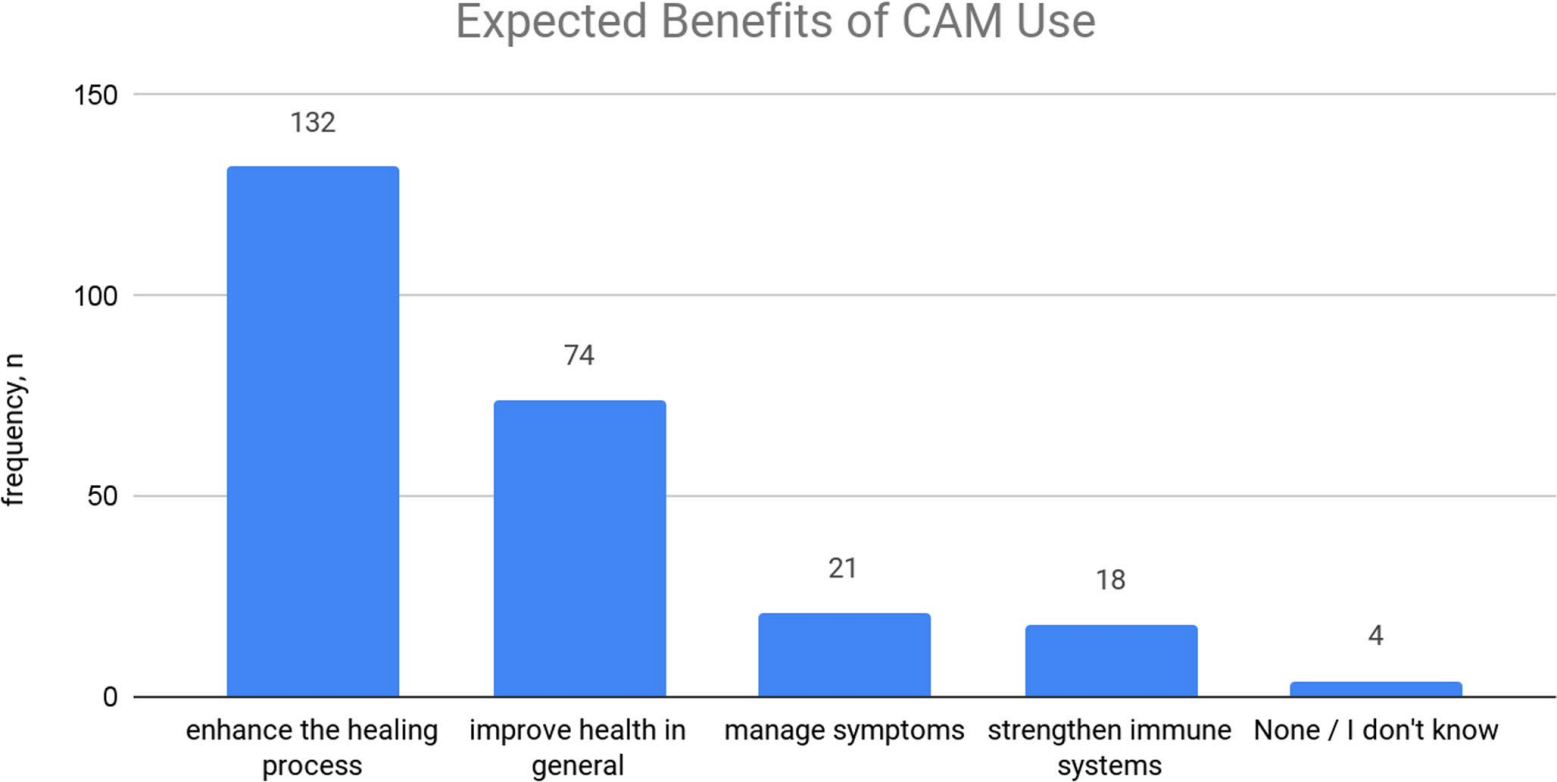
Perceptions of CAM benefits reported by CAM Users

Patterns of CAM practices amongst fracture patients varied (Figure. 2) with fenugreek being the most commonly used herbal medicine by 142 patients (81%) of CAM users followed by Mung bean by 79 users (45%). The majority of CAM users (60.6%) reported using CAM daily followed by patients who used them few times a week (25.7%) and few times a month (8%). The primary motives for CAM practices were mostly to maintain their health 129 (74.6%), followed by preventing future illness 37 (21.4%) then, surprisingly, their doctor’s recommendation 18 (10.4%). There are common perceptions among CAM users about their benefits (Fig. 3) which involve enhancing fracture healing process 132 (76.7%), improving general health 74 (43%) and managing symptoms 21 (12.2%). In terms of the subjective experienced benefits by CAM users, 107 (61.50%) felt improvement in physical health, 75 (43.10%) reported relief of bothersome symptoms, while 15 (8.6%) experienced mental health benefits. On the other hand, 41 (23.60%) CAM users did not feel any benefits after using them. No adverse effects were encountered or reported by most CAM users 157 (90%). Nevertheless, 16 users (9%) mentioned having new physical health issues which included symptoms like weight gain, joints pain, gastrointestinal symptoms and allergies, while 2 patients (1%) experienced worsening of their pre-existing bothersome symptoms. While these associations provide useful insights, the study did not perform a multivariable logistic regression analysis to identify independent predictors of CAM use. Such modeling would strengthen the robustness of findings, and future studies are recommended to incorporate this approach.

**Fig. 4:**
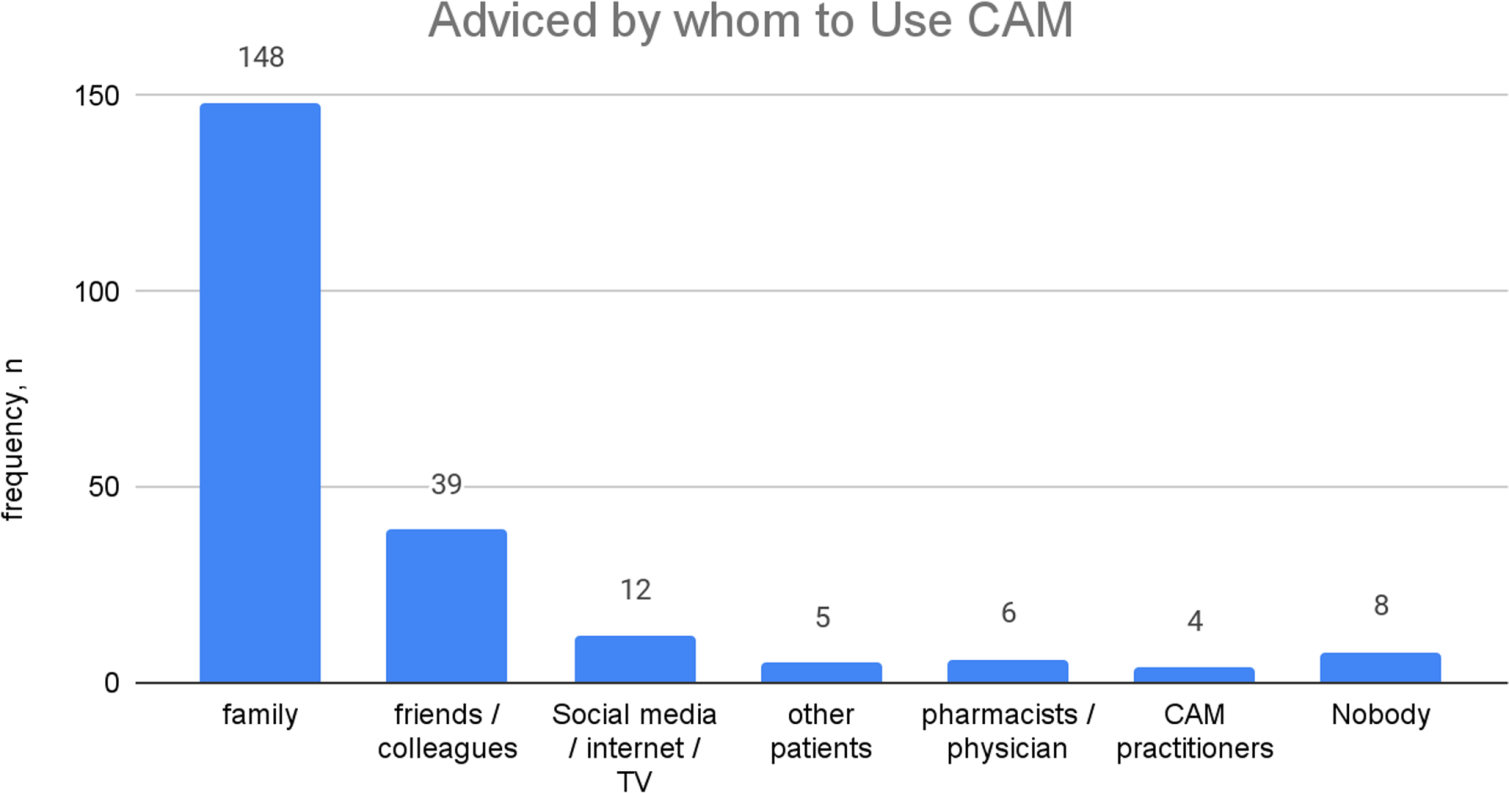
Factors associated with CAM use among fracture patients

When CAM users were inquired as to who influenced them to consider CAM usage (Fig. 4), Family plays a considerable role in influencing the majority 148 (84.60%), followed by friends and colleagues 39 (22.30%) or online or media resources (Social media, internet, TV) in 12 (6.90%). Similarly, 155 of CAM users (88%) are willing to influence and recommend other fracture patients to consider using CAM after their experience.

Interestingly, only 84 CAM users (47.7%) have discussed usage of CAM with their physician. Among those, 40 patients (47.6%) reported no objection by their physicians regarding CAM, while 37 (44.0%) noted that their physicians were neutral and 1 patient (1.1%) was discouraged by his physician in this regard. Conversely, 92 patients (52.2%) did not disclose CAM usage to their treating doctor and the most common reported reason for not disclosing it was attributed to “physician did not ask about it” in 77 patients (70%) while 17 (15.5%) thought it was unnecessary. The most favoured source for acquiring CAM remedies were traditional herbal shops as reported by 160 patients (91%). Other less frequently reported sources of CAM include homemade as well as authorized facilities such as pharmacies and certified online shops, each accounting for 18 (10%).

## Discussion

Complementary and alternative medicine (CAM) use among patients with musculoskeletal conditions, bone fractures particularly, is frequently encountered in clinical practice mostly due to cultural and spiritual factors [12, 13].

The prevalence, perceptions and various practices of CAM usage among fracture patients presents a compelling area for investigation. Our study revealed a high prevalence of CAM usage (49.4%) among fracture patients in Saudi Arabia in comparison to findings of previous studies from Germany and Canada with reported prevalence of 40% and 35%, respectively, suggesting that regional variations may significantly influence the acceptance and utilization of CAM [14, 15].

Certain factors probably associated with patient behavior towards CAM usage were investigated in our study. Our results have shown a male patient’s predominance in using CAM in 58.5% of our sample. This is relatively consistent with the results of a previous study although gender difference in both studies has not shown to be statistically significant [14]. Furthermore, age played a significant role in CAM utilization amongst our patients. An article discussing population characteristics in regards to CAM usage has shown that usage of CAM is higher among older population [16]. They reported that factors such as cultural and social factors play a role for an older majority of patients to consider CAM in their treatment regimen. Almost half of our sample of CAM users have an educational level higher than a high school diploma. A previous study found that higher educational level was found to be a significant factor in predicting CAM usage, with higher levels of education being associated with a greater likelihood of using CAM. Specifically, individuals with more than a high school education were significantly more likely to use CAM compared to those with a high school education or less [17]. However, our study did not demonstrate a similar correlation of educational level with CAM practices. In terms of income, 46.6% of the population that were willing to disclose their income had an income of approximately 2,130 USD. According to Bazargan et al. [17]. financial strain was linked to a higher likelihood of turning to alternative healthcare as a replacement for conventional medical treatment. This could possibly be attributed to less educational level or limited access to specialized healthcare facilities.

About two thirds (62.5%) of CAM users reported a fracture healing period of more than 3 months. People with delayed union and prolonged recovery time tend to explore various treatment modalities alongside conventional options when compared to people with a short duration of healing. Patients with lower extremity fractures utilized CAM practices more frequently than those with upper extremity fractures (57.9% and 36.8% respectively). This may be explained by the fact that lower extremity fractures usually take longer time for healing and rehabilitation when compared to upper extremity fractures [14]. In line with this, We found that there is a statistically significant correlation between disability/dependence and CAM usage. Furthermore, patients’ symptoms, particularly pain, was a contributing factor for higher CAM utilization in 41% of our sample. Vyas et al. [18] have discussed the analgesic effects of fenugreek, and how they benefit patients with chronic pain. Acupuncture, is another CAM modality that has been studied for its potential benefits for chronic musculoskeletal pain [19].

It is noteworthy that 50.6% of the population did not use nor increase usage of CAM after they sustained fracture. This could be due to the fact that CAM usage is not frequently encouraged by physicians and not being a standard practice for fracture treatment. A 15-year systematic review discussing acceptance and use of complementary and alternative medicine among medical specialists has mentioned that some physicians remain skeptical of CAM due to limited knowledge and training, as well as the absence of high-quality evidence proving the effectiveness of CAM treatments [9]. However, 39.3% of the population have increased their use of CAM after sustaining a fracture, indicating that there is a significant portion of the population already using CAM with their baseline health. Fenugreek followed by Mung bean were found to be the most commonly used CAM in our sample. There is cultural belief in the Middle East that these CAM modalities may enhance fracture healing and the recovery process. According to a report by Aldhilan et al. [16], It’s commonly believed in the Middle East that fenugreek aids in fracture healing. Studies have shown that, when used as a CAM, fenugreek can stimulate osteogenesis, reduce inflammation, and potentially improve bone healing [20]. Furthermore, various commonly practiced CAM modalities are yet to undergo robust trials to investigate their clinical benefits and evaluate the safety profile in the long term. The majority of CAM users (61.5%) have claimed experiencing improvements in physical health with no reported adverse effects in 90% of our sample. Despite health education efforts, patients often practice CAM modalities on their own without seeking expert medical advice and only 10% of participants in our study claim that CAM usage was encouraged by their physician. Only 47.7% of patients have disclosed their CAM usage to their physicians and 70% attribute lack of disclosure to not being asked about CAM by their treating physicians. Failure to communicate CAM use may lead to harmful drug–herb interactions, interference with peri-operative management and reduced medication efficacy [21–23], highlighting the importance of health education, enhancing awareness of the potential risks of CAM practices, and patient-centered communication.

Although our study provides valuable insights, it could be subject to potential biases which are sometimes associated with observational cross-sectional survey studies. For instance, non-response bias, where the non-respondents cohort might differ systematically from respondents in their CAM use. Nonetheless, the potential impact of non-respondents on the results is likely minimal given 75.7% response rate of our study, in addition, the majority of non-respondents were from the virtual clinics’ list who did not answer the repeated phone calls rather than due to refusal to participate.Another possible bias is volunteering bias, in which participants may be more inclined to volunteer to participate if they are interested in the subject of the study. Although the relatively high prevalence of CAM usage among fracture patients in our sample may be partly due to this bias, our findings are consistent with a meta-analysis study in Saudi Arabia [24] Additionally, other biases associated with the subjective nature of self-reported data such as reporting bias and recall bias are less likely due to the relatively short period between the injury and survey time. While we did not assess comorbidities which could affect CAM use (e.g. osteoporosis and diabetes mellitus), nevertheless, the relatively young median age of our sample (29.0 ± 21.0 years) probably suggests minimal impact of possible associated comorbidities on the observed outcomes.

The objective metrics to assess the impact on fracture healing and clinical benefits of CAM use are not feasible due to the subjective nature of our study and the wide variety of non-standardized CAM practices among our participants. Although the primary purpose of this study was to evaluate the prevalence and perceptions of CAM practices among fracture patients, further studies are needed to investigate the clinical implications, safety profile, adverse events as well as possible benefits and risks of common CAM practices.

## Conclusions

Our study shed a light on the high prevalence of CAM usage among fracture patients (49.4%), with Fenugreek being the most popular. Most influencing factors for CAM practices were investigated including demographic, socioeconomic as well as fracture-related factors. Despite its high prevalence of CAM utilization, it is not often discussed with the treating physicians. Hence, increased physician awareness and enhanced patient communication about CAM practices are essential. Further future studies are recommended on strategies to enhance physician–patient communication about CAM along with the safety profile of various CAM practices, possible risks and presumptive benefits of CAM modalities, and the influence of culture on CAM use amongst different populations.

## Data Availability

The data presented in this study are available on request from the corresponding author.

## References

[1] Barnes PM, Bloom B, Nahin RL. Complementary and alternative medicine use among adults and children: United States, 2007. PsycEXTRA Dataset [Internet]. 2008 [cited 2025 Jan 27]. Available from: https://www.researchgate.net/publication/24272451_Complementary_and_Alternative_Medicine_Use_Among_Adults_and_Children_United_States_2007 19361005

[2] Complementary. Alternative, or Integrative Health: What’s in a name? | NCCIH [Internet]. [cited 2024 Dec 24]. Available from: https://www.nccih.nih.gov/health/complementary-alternative-or-integrative-health-whats-in-a-name

[3] What are complementary and alternative therapies? | Cancer Research UK [Internet]. [cited 2024 Dec 24]. Available from: https://www.cancerresearchuk.org/about-cancer/treatment/complementary-alternative-therapies/about/difference-between-therapies

[4] Buran-Omar AP, Alaban AG. Integrating Al-Hijamah into the healthcare system in Saudi Arabia: hospital staff’s perception, possible use, and acceptability. Complement Med Res. 2022;29(3):228–34. 10.1159/000522469. 35134806 10.1159/000522469

[5] Bischoff-Ferrari HA, Willett WC, Wong JB, Giovannucci E, Dietrich T, Dawson-Hughes B. Fracture prevention with vitamin D supplementation: a meta-analysis of randomized controlled trials. JAMA [Internet]. 2005 [cited 2024 Dec 24]:293(18):2257–64. Available from: https://www.ncbi.nlm.nih.gov/books/NBK71740/10.1001/jama.293.18.2257 15886381

[6] Bischoff-Ferrari HA, Willett WC, Orav EJ, Lips P, Meunier PJ, Lyons RA et al. A pooled analysis of vitamin D dose requirements for fracture prevention. N Engl J Med [Internet]. 2012 [cited 2024 Dec 24]: 367(1):40–9. Available from: https://pubmed.ncbi.nlm.nih.gov/22762317/10.1056/NEJMoa1109617 22762317

[7] Gunawan G, Upoyo AS, Triyanto E, Ramawati D, Sari Y. Complementary therapy to reduce pain intensity to treat chronic pain in fracture patients: a systematic review. Malahayati Int J Nurs Health Sci. 2024;7(6):701–8.

[8] Yazdi N, Salehi M, Ghorat F, Hashempur MH. Exploring traditional and complementary medicine approaches for fractured bones: A systematic review. Galen Med J. 2024;13:e3227. 10.31661/gmj.v13i.3227 PMC11827764 42038142

[9] Aldhilan MM, Abdel-Wanis ME. We needs to look more into possible benefits of fenugreek for bone health: a short commentary. [cited 2024 Dec 24]. Available from: http://creativecommons.org/licenses/by/4.0/

[10] Alsanad S, Aboushanab T, Khalil M, Alkhamees OA. A descriptive review of the prevalence and usage of traditional and complementary medicine among Saudi diabetic patients. Scientifica. 2018;2018:1DUUMMY. 10.1155/2018/6303190 PMC6136479 30228928

[11] Tangkiatkumjai M, Boardman H, Walker DM. Potential factors that influence usage of complementary and alternative medicine worldwide: a systematic review. BMC Complementary Medicine and Therapies 2020 20:1 [Internet]. 2020 [cited 2025 Jan 27]:20(1):1–15. Available from: https://bmccomplementmedtherapies.biomedcentral.com/articles/10.1186/s12906-020-03157-2 10.1186/s12906-020-03157-2 PMC7686746 33228697

[12] Alsaleh K, Alkhenizan Z, Aldossari A, Alammari A, Dakhil A, Bin, Alzakri A, Patients. ’ attitudes toward alternative medicine as a treatment for musculoskeletal conditions: one center’s experience. Journal of Nature and Science of Medicine [Internet]. 2022 [cited 2024 Dec 24]:5(1):7–10. Available from: https://journals.lww.com/jnsm/fulltext/2022/05010/patients__attitudes_toward_alternative_medicine_as.3.aspx

[13] Alnaimat F, Alduraidi H, Alhafez L, Raddad LA, Haddad BI, Hamdan M et al. Rates, patterns, and predictors of complementary medicine use among patients with musculoskeletal diseases. PLoS One [Internet]. 2023 [cited 2025 Jan 27]:18(6):e0287337. Available from: https://pmc.ncbi.nlm.nih.gov/articles/PMC10289458/10.1371/journal.pone.0287337 PMC10289458 37352251

[14] Kilper A, Müller A, Huber R, Reimers N, Schütz L, Lederer AK. Complementary medicine in orthopaedic and trauma surgery: a cross-sectional survey on usage and needs. BMJ Open [Internet]. 2020 [cited 2024 Dec 25]:10(9):e037192. Available from: https://pmc.ncbi.nlm.nih.gov/articles/PMC7477982/10.1136/bmjopen-2020-037192 PMC7477982 32895280

[15] Sprague S, Lutz K, Bryant D, Farrokhyar F, Zlowodzki M, Bhandari M. Complementary and alternative medicine use in patients with fractures. Clin Orthop Relat Res [Internet]. 2007 [cited 2025 Jan 27];463:173–8. Available from: https://journals.lww.com/clinorthop/fulltext/2007/10000/complementary_and_alternative_medicine_use_in.27.aspx 17960679

[16] Analgesic and anti-inflammatory activities of Trigonella foenum-. graecum (seed) extract - PubMed [Internet]. [cited 2024 Dec 24]. Available from: https://pubmed.ncbi.nlm.nih.gov/19051589/19051589

[17] Bazargan M, Norris KC, Bazargan-Hejazi S, Akhanjee L, Calderón JL, Safvati S et al. Alternative healthcare use in the under-served population. Ethn Dis. 2005. 16259473

[18] Phutrakool P, Pongpirul K. Acceptance and use of complementary and alternative medicine among medical specialists: a 15-year systematic review and data synthesis. Syst Rev [Internet]. 2022 [cited 2024 Dec 24]:11(1). Available from: https://pubmed.ncbi.nlm.nih.gov/35027078/10.1186/s13643-021-01882-4 PMC8759198 35027078

[19] Ernst E. Acupuncture–a critical analysis. J Intern Med [Internet]. 2006 [cited 2025 Jan 27]: 259(2):125–37. Available from: https://pubmed.ncbi.nlm.nih.gov/16420542/10.1111/j.1365-2796.2005.01584.x 16420542

[20] Aldhilan MM, Abdel-Wanis ME, Aldhilan M, Abdel-Wanis ME. The healing callus-promoting effect of fenugreek in a humerus shaft fracture: a case report. Cureus [Internet]. 2023 [cited 2025 Jan 27];15(12). Available from: https://www.cureus.com/articles/214084-the-healing-callus-promoting-effect-of-fenugreek-in-a-humerus-shaft-fracture-a-case-report 10.7759/cureus.50519 PMC10720763 38098736

[21] Skinner CM, Rangasami J. Preoperative use of herbal medicines: A patient survey. Br J Anaesth. 2002;89(5):792–5. 12393786

[22] Elvir Lazo OL, White PF, Lee C, Cruz Eng H, Matin JM, Lin C, et al. Use of herbal medication in the perioperative period: potential adverse drug interactions. J Clin Anesth. Volume 95. Elsevier Inc.; 2024. 10.1016/j.jclinane.2024.111473 38613937

[23] Izzo AA, Ernst E. Interactions between herbal medicines and prescribed drugs: an updated systematic review. Drugs. 2009;69:1777–98. 19719333 10.2165/11317010-000000000-00000

[24] Alsanad SM. A comprehensive look at complementary and alternative medicine (CAM) in Saudi arabia: a meta-analysis study. Health Science Reports. Volume 8. John Wiley and Sons Inc; 2025. 10.1002/hsr2.70491 PMC12006920 40256149

